# Microbial metaproteome data from decayed beech dead wood

**DOI:** 10.1016/j.dib.2020.105285

**Published:** 2020-02-14

**Authors:** Lydia Kipping, Nicholas Holzscheck, Florian Maurer, Sarah Muszynski, Matthias Noll, Nico Jehmlich

**Affiliations:** a Department of Molecular Systems Biology, Helmholtz Centre for Environmental Research GmbH - UFZ, Permoserstraße 15, 04318, Leipzig, Germany; b Coburg University of Applied Sciences and Arts, Institute for Bioanalysis, Friedrich-Streib-Straße 2, 96450, Coburg, Germany

**Keywords:** Dead wood decay, Metaproteomics, Protein extraction, Fungal-bacterial community

## Abstract

Wood-decomposition in terrestrial ecosystems is a very important process with huge ecologic consequences. This decomposition process is a combination of biological respiration, leaching and fragmentation, mainly triggered by organismic activities. In order to gain a deeper insight into these microbial communities and their role in deadwood decay, we used metaproteomics. Metaproteomics is an important tool and offers the ability to characterize the protein complement of environmental microbiota at a given point in time. In this dataset, we provide data of an exemplary beech wood log and applied different extraction methods to provide the proteome profile of beech dead wood and their corresponding fungal-bacterial community.

**Table:** Specifications Table

Subject	Environmental Science; Genetics, Genomics and Molecular Biology; Proteomics
Specific subject area	Metaproteomics of dead wood microbial communities
Type of data	1) Mass spectrometry data (*.raw) 2) Search output data (*.msf) 3) Figures (PowerPoint files)
How data were acquired	Q Exactive HF mass spectrometer (Thermo Scientific) coupled with the TriVersa NanoMate (Advion Biosciences, Norwich, UK)
Data format	1) Mass spectrometry data (*.raw) 2) Search output data (*.msf) 3) Figures (PowerPoint files)
Parameters for data collection	Beech wood log was collected in spring 2017 from a Biodiversity Exploratory in Germany
Description of data collection	Microbial proteins were isolated of a beech dead wood sample, proteolytic cleaved using trypsin and subsequently analyzed by LC-MS/MS
Data source location	Leipzig, Germany
Data accessibility	Project Name: Microbial metaproteome data from decayed beech dead wood Repository name: ProteomeXchange Data identification number: PXD016801 Data link: https://www.ebi.ac.uk/pride/archive/projects/PXD016801

**Table:** 

**Value of the Data** • Metaproteome data of beech dead wood provide valuable insight into fungal-bacterial communities. • Metaproteome provides the basis for a deeper mechanistic understanding of dead wood decomposition processes and their relation to biodiversity. • These data are valuable to serve for comparative ecological studies to gain further insights in forest ecosystems and the role of dead wood.

## 1 Data description

This data set presents the construction of the microbial communities in coarse woody debris (CWD). CWD is an important structural component of the forest ecosystem and influences a large number of ecosystem functions [1,2]. In recent years, the attention focused on fungi and insects as members of the micro-ecosystem in CWD and their important role at the process of deadwood decay. However, beside fungi and insects, diverse bacterial communities participate also in wood degradation process, in oxic as well in anoxic conditions [3,4]. The fungal-bacterial community in dead wood has previously not been investigated by metaproteomics. In order to obtain the highest amount of reliable information, several methods of sample preparation were tested. Therefore, a beech wood log was ground to fine powder, divided into eight samples, treated with different extractions procedures and measured with LC-MS/MS.

**Fig. 1:**
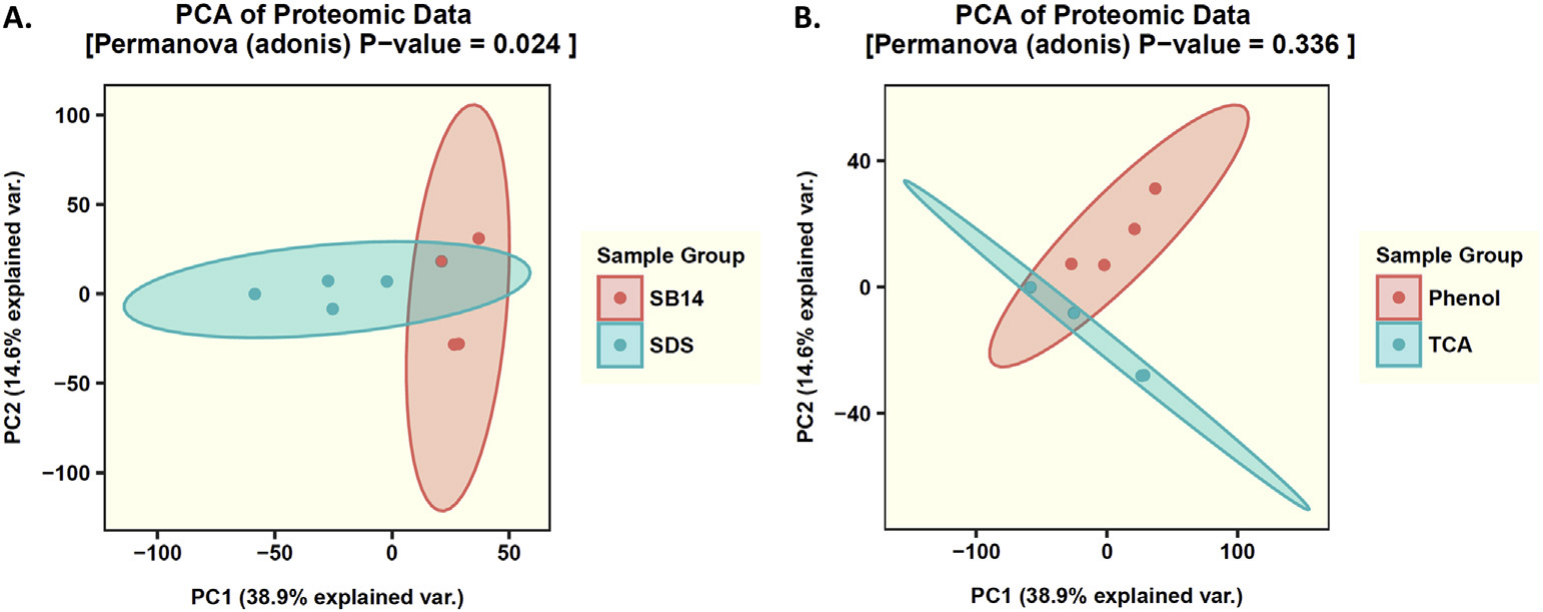
Principal component analysis (PCA) of different protein extractions methods for beech dead wood. PCA shows the dissimilarities for the tested samples, which were treated as follows, first with the cell lysis buffer sodium dodecyl sulfate (SDS) or sulfobetain-14 (SB-14), and then with the protein extraction methods based on phenolic buffer (Phenol) and precipitation with ammonium acetate in methanol or direct precipitation with trichloroacetic acid (TCA) in acetone. Each group contains four replicates.

**Fig. 2:**
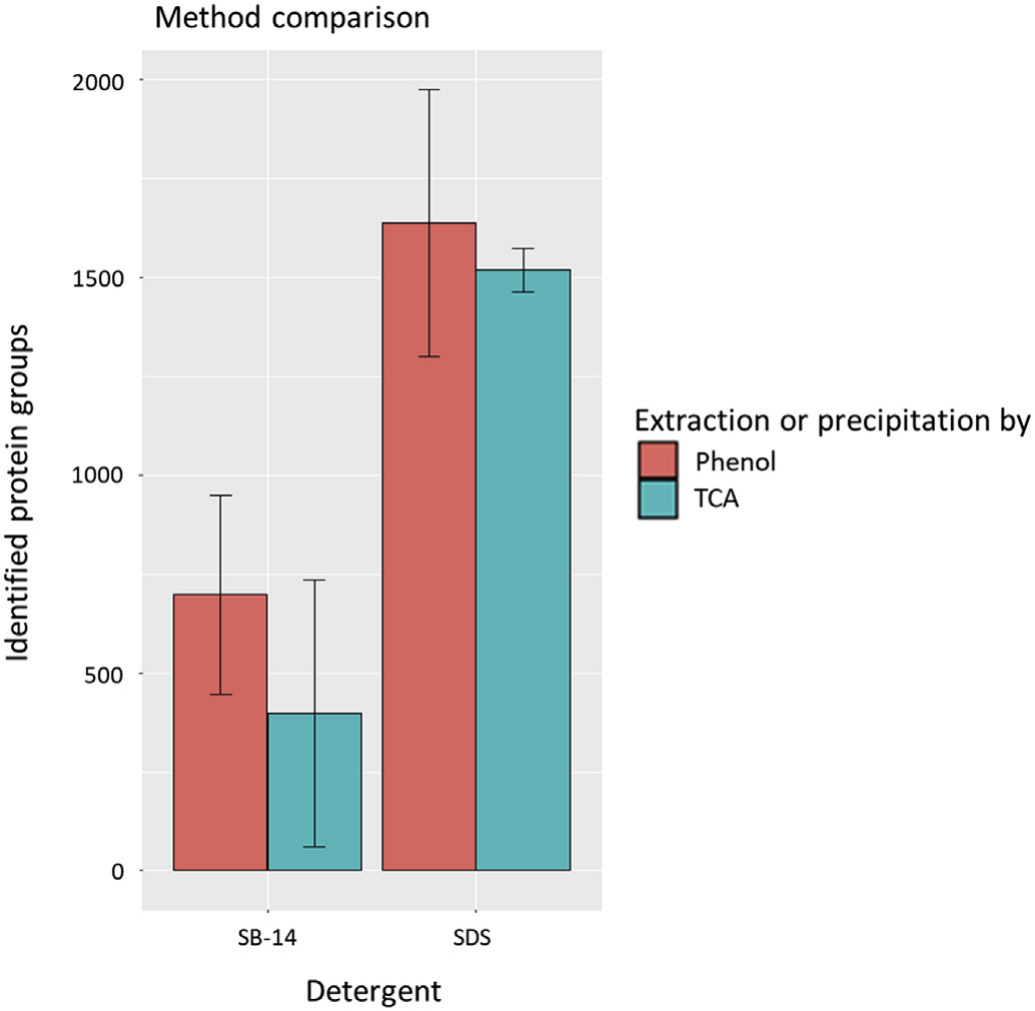
Count of identified protein groups depending on the different protein extraction methods from beech dead wood. The cell lysis was performed either with the detergents sodium dodecyl sulfate (SDS) or sulfobetain-14 (SB-14), and compared with the following methods, the protein extraction methods based on phenolic buffer (Phenol) and precipitation with ammonium acetate in methanol or direct precipitation with trichloroacetic acid (TCA) in acetone. Each combination represents two replicates.

**Fig. 3:**
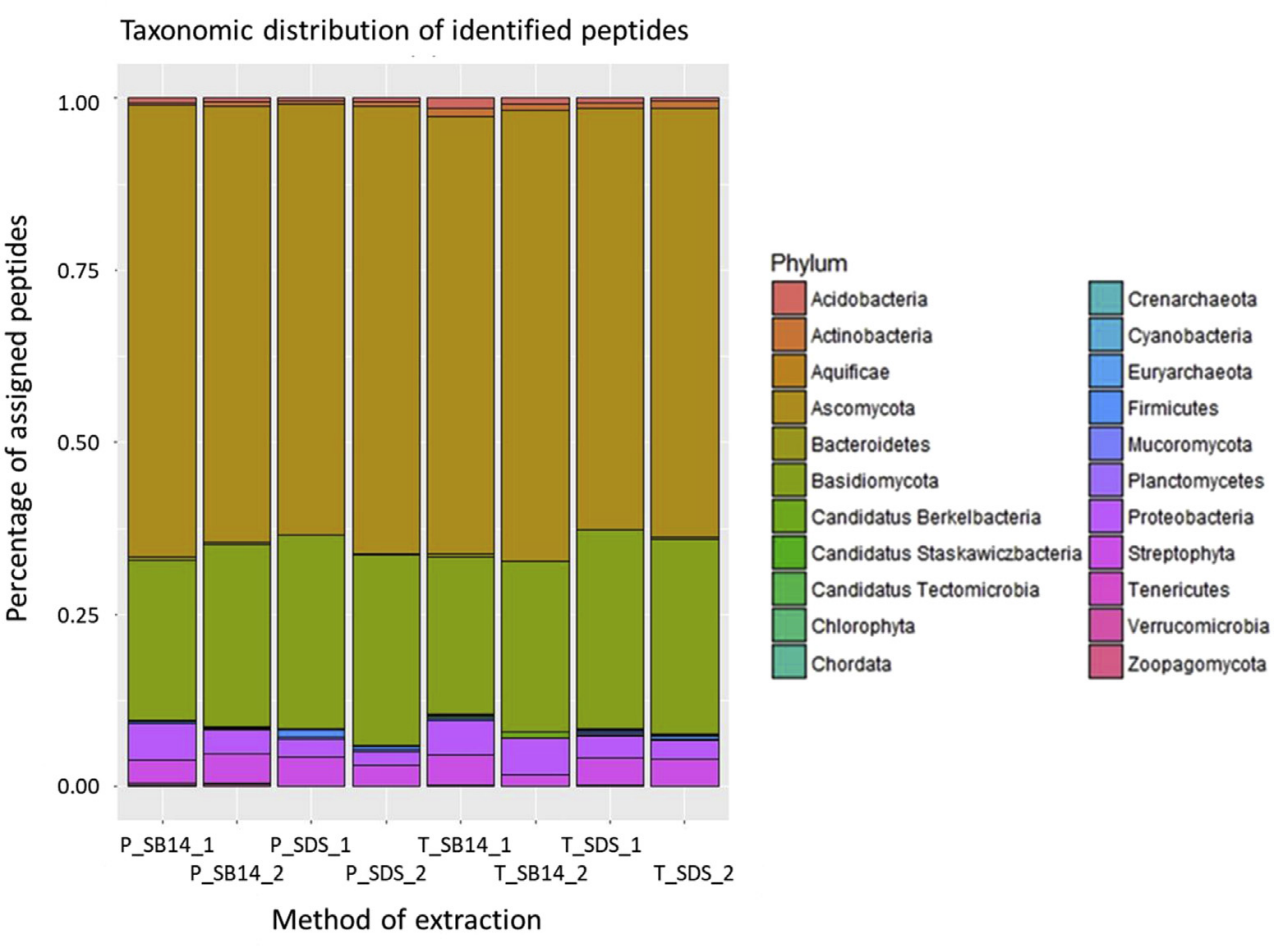
Phylogenetic resolution of identified peptides of beech dead wood. The sample preparation were carried out with the lysis buffer sodium dodecyl sulfate (SDS) or sulfobetain-14 (SB14) in combination with phenol (P) extraction plus precipitation with ammonium acetate in methanol or direct precipitation with trichloroacetic acid (TCA) in acetone.

For this purpose, the steps of cell lysis and protein extraction were investigated. The protein lysis buffers contained two different detergents, either sodium dodecyl sulfate or sulfobetain-14. The protein extraction was performed by either adding phenolic buffer and precipitation with ammonium acetate in methanol or direct precipitation with trichloroacetic acid in acetone. These samples were analyzed with mass spectrometers and graphically evaluated with *R*. Fig. 1A and B showed the results of the principal component analysis (PCA). The number of identified protein groups per extraction procedure are shown (Fig. 2). For the phenol extraction-based method in combination with sodium dodecyl sulfate buffer the highest amount of protein groups was determined. In addition, the taxonomic analysis of all samples are provided (Fig. 3). The most abundant fungi are Ascomycota and Basidiomycota, for bacteria Proteobacteria and Tenericutes has been detected.

## 2 Experimental design, materials, and methods

### 2.1 Study area and sampling

In the Biodiversity Exploratory Hainich-Dün, Germany (for details, see Ref. [5]), we collected in spring 2017 from a beech log, dead wood chips for extraction testing as outlined previously [6].

### 2.2 Protein extraction and sample preparation

The wood chips was roughly sliced with a cutting blade and then milled with added liquid nitrogen in the analytical mill (IKA A11 basic, IKA grinder) into fine powder. 1 g of powder were divided on eight 15 ml tube and used for testing for the different protein extraction protocols; cell lysis with sodium dodecyl sulfate (SDS) buffer (2% (w/v) SDS, 50 mM DTT, 50 mM EDTA, 1 mM PMSF, 100 mM Tris) or sulfobetain-14, and additional phenol extraction or trichloroacetic acid (TCA)-acetone (10% (w/v) TCA, 2-Mercaptoethanol, acetone) precipitation. Two samples were analyzed for each combination. The sample preparation is indicated in the following flow chart:

**Figure:**
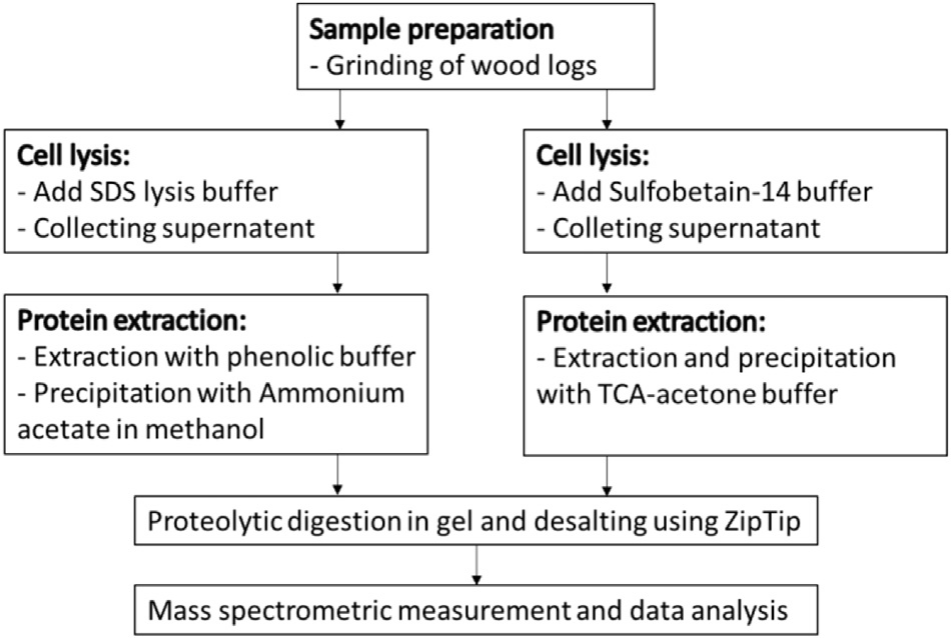

#### 2.2.1 Cell lysis

5 ml lysis buffer (SDS or Sulfobetain-14 buffer), 10 glass beads (2,85–3,45 mm diameter, CarlRoth) and 3 scoops of zirconium beads (0,1 mm diameter, Biospec.) were added to samples, shaken for 30 minutes at room temperature and disrupted by FastPrep (3 × 1 min, 5.5 ms^−1^, MP Biomedicals). After centrifugation (7200 x g, 10 min, 4 °C), the supernatant were transferred to 50 ml tubes and the proteins were extracted.

#### 2.2.2 Phenolic extraction

For phenolic protocol, 5 ml extraction buffer were added, shaken for 30 minutes at room temperature and phases separated by centrifugation (7200 x g, 10 min, 4 °C). Afterwards, the phenolic phase were transferred to a new 50 ml tube. This procedure of extraction buffer and centrifugation were repeated three times, all phenolic phases were collected and pooled in one tube. The extracted proteins were precipitated over night at −20 °C with precipitation solution (4 fold of 100 mM Ammonium acetate in methanol). The protein pellets were harvested by centrifugation (7200 x g, 10 min, 4 °C), washed three times with 1 ml precipitation solution and finally once with 1 ml ice-cold acetone. After supernatant discard, the dried pellets were dissolved in SDS sample buffer and proteolytic digested in gels.

#### 2.2.3 TCA protein extraction

For TCA extraction 20 ml (4 fold) of TCA-acetone buffer were added, mixed by inverting and precipitated over night at −20 °C. The protein pellets were harvested by centrifugation (7200 x g, 10 min, 4 °C), re-suspended in 800 μl TCA-Acetone buffer and transferred to 2 ml tubes. The suspension was again centrifuged (16,000 x g, 10 min, 4 °C), the supernatant was discarded and the protein pellet was rinsed twice with 1 ml ice-cold acetone.

#### 2.2.4 Proteolytic digestion and desalting

For all extraction methods, the dried pellets were proteolytic digested in gel. Therefore, the pellets were dissolved in SDS sample buffer (2% w/v SDS, 2 mM beta-mercaptoethanol, 4% v/v glycerol, 40 mM Tris–HCl pH 6.8, 0.01% (w/v) bromophenol blue), heated to 90 °C for 4 minutes and separated by SDS polyacrylamide gel electrophoresis. Proteins were stained in gel with Coomassie G-250 (Merck). Then, the gel was cut into small pieces (band per samples), detained, dehydrated and proteolytically cleaved overnight at 37 °C with trypsin (Promega). The digested peptides were extracted and desalted using ZipTip-μC18 tips (Merck Millipore, Darmstadt, Germany). Following, the peptide lysates were re-suspended in 0.1% formic acid and injected to liquid chromatography mass spectrometry (LC-MS/MS).

### 2.3 Mass spectrometric measurement

The volume of 5 μl re-suspended peptides were injected into nanoHPLC (UltiMate 3000 RSLCnano, Dionex, Thermo Fisher Scientific), trapped on a C18-reverse phase trapping column (C18 PepMap100, 300 μm × 5 mm, particle size 3 μm, Thermo Fischer Scientific) and followed by separation on a C18-reverse phase analytical column (Acclaim PepMap® 100, 75 μm × 25 cm, particle size 3 μm, nanoViper, Thermo Fischer Scientific). Both columns were tempered to 40 °C at all times. Mass spectrometry of eluated peptides were analyzed on a Q Exactive HF mass spectrometer (Thermo Fisher Scientific, Waltham, MA, USA) coupled to a TriVersa NanoMate source (Advion, Ltd., Harlow, UK) in LC chip coupling mode. The LC Gradient, ionization and MS settings were described in detail [7].

### 2.4 Data analysis

Data resulting from LC-MS/MS experiments were analyzed using the Proteome Discoverer (v.1.4, Thermo Fischer Scientific, Waltham, MA, USA) using SEQUEST HT. As database the protein-coding sequences of archaea, bacteria, fungi and viridiplantae were downloaded from UniProt_non_redundant_07_2017 (http://www.uniprot.org/), combined in one protein-coding sequence database (*.fasta). Search settings were set to trypsin (Full), max. missed cleavage: 2, precursor mass tolerance: 10 ppm, fragment mass tolerance: 0.02 Da.

For visualization, the principal component analysis (PCA) was analysis with *R* (RStudio v1.2.5019) and applied as classical means of dimensionality reduction and visualization of multivariate data. PCA was assessed of log-transformed and normalized protein abundance profiles along the time scale. Additional, Unipept (https://unipept.ugent.be/) and *R* (RStudio v1.2.5019) was used for taxonomic analysis.
